# The Effects of Natural and Anthropogenic Microparticles on Individual Fitness in *Daphnia magna*

**DOI:** 10.1371/journal.pone.0155063

**Published:** 2016-05-13

**Authors:** Martin Ogonowski, Christoph Schür, Åsa Jarsén, Elena Gorokhova

**Affiliations:** 1 Department of Environmental Science and Analytical Chemistry, Stockholm University, Stockholm, Sweden; 2 AquaBiota Water Research, Stockholm, Sweden; VIT University, INDIA

## Abstract

Concerns are being raised that microplastic pollution can have detrimental effects on the feeding of aquatic invertebrates, including zooplankton. Both small plastic fragments (microplastics, MPs) produced by degradation of larger plastic waste (secondary MPs; SMPs) and microscopic plastic spheres used in cosmetic products and industry (primary MPs; PMPs) are ubiquitously present in the environment. However, despite the fact that most environmental MPs consist of weathered plastic debris with irregular shape and broad size distribution, experimental studies of organism responses to MP exposure have largely used uniformly sized spherical PMPs. Therefore, effects observed for PMPs in such experiments may not be representative for MP-effects *in situ*. Moreover, invertebrate filter-feeders are generally well adapted to the presence of refractory material in seston, which questions the potential of MPs at environmentally relevant concentrations to measurably affect digestion in these organisms. Here, we compared responses to MPs (PMPs and SMPs) and naturally occurring particles (kaolin clay) using the cladoceran *Daphnia magna* as a model organism. We manipulated food levels (0.4 and 9 μg C mL^-1^) and MP or kaolin contribution to the feeding suspension (<1 to 74%) and evaluated effects of MPs and kaolin on food uptake, growth, reproductive capacity of the daphnids, and maternal effects on offspring survival and feeding. Exposure to SMPs caused elevated mortality, increased inter-brood period and decreased reproduction albeit only at high MP levels in the feeding suspension (74% by particle count). No such effects were observed in either PMP or kaolin treatments. In daphnids exposed to any particle type at the low algal concentration, individual growth decreased by ~15%. By contrast, positive growth response to all particle types was observed at the high algal concentration with 17%, 54% and 40% increase for kaolin, PMP and SMP, respectively. When test particles comprised 22% in the feeding suspension, both MP types decreased food intake by 30%, while kaolin had no effect. Moreover, SMPs were found to homoaggregate in a concentration-dependent manner, which resulted in a 77% decrease of the ingested SMPs compared to PMPs. To better understand MP-processing in the gut, gut passage time (GPT) and evacuation rate of MPs were also assayed. SMPs and PMPs differed in their effects on daphnids; moreover, the particle effects were dependent on the MP: algae ratio in the suspension. When the MP contribution to the particle abundance in the medium changed from 1 to 4%, GPT for daphnids exposed to SMPs increased 2-fold. Our results suggest that MPs and, in particular, SMPs, have a greater capacity to negatively affect feeding in *D*. *magna* compared to naturally occurring mineral particles of similar size. Moreover, grazer responses observed in experiments with PMPs cannot be extrapolated to the field where SMPs dominate, because of the greater effects caused by the latter.

**Highlights**

- Effects of exposure to MPs on individual fitness in *Daphnia* were studied.
- Lowered feeding and reproduction were observed at high MP levels.
- Secondary MPs are more harmful than primary MPs.
- Secondary MPs increase gut passage time and form aggregates in the gut.

## 1. Introduction

Plastic debris is increasing in aquatic environments, and its potential impact has raised environmental concerns. The major concerns have been related to food web effects, due to accidental ingestion of plastic materials that has been documented for a number of species. In some surveys of seabirds, fish, turtles and mammals, the plastic items have been reported to occur in nearly all specimens examined [1,2]. The plastic ingestion has been implicated in mechanical disturbance of the gastrointestinal tract and, in extreme cases, starvation.

In addition to the large plastic debris, microplastics (MPs, <5 mm) are recognized as emerging contaminants in freshwater and marine ecosystems. Primary MPs (so-called plastic pellets) are manufactured and used in a variety of products (e.g., cosmetics and abrasives; [3] and enter aquatic environments mostly via water treatment plants. Secondary microplastics are microscopic fragments derived from the breakdown of larger plastic debris. Evidence is now accumulating that <1 mm plastic pellets, fibers and irregularly shaped fragments [4] are ingested by organisms at lower trophic levels, such as deposit feeders [5,6] and filterers [7,8]. Moreover, it has been proposed that, similar to the larger plastic particles, MPs may accumulate in the digestive tracts upon ingestion, decrease actual food intake, growth and, possibly, in the long run, starvation and death [9–11]. Furthermore, the offspring produced by nutritionally challenged mothers may have poor nutritional status already from birth and suffer lower feeding activity and higher mortality. In daphnids, for example, malnutrition of mothers has strong negative effects on offspring quality [12,13], and, in theory, the presence of MP or other inert particles in the diet may have similar consequences.

In aquatic environments, however, suspension- and filter feeders are well adapted to feed on small particles of varying palatability. Therefore, the likelihood of gut obstruction by MPs in these invertebrates is questionable. In fact, MPs in the size range 1–20 μm have commonly been used to study various aspects of zooplankton feeding ecology, such as size selectivity and various factors affecting ingestion rate [14–17]. Moreover, in this size range, MPs do not stand out from other particles, such as clay, sand, and refractory organic matter that are ubiquitous in natural seston. Clay and other mineral particles are known to be ingested by a wide range of zooplankters (cladocerans: [18–20], copepods: [18,21], rotifers: [22] and ciliates: [23]) without any observable negative effects of the particles *per se*. It is also recognized that the increasing contribution of non-palatable particles in the food suspension may decrease food intake [18,24] and, thus, lower overall productivity [25,20], but see Rellstab and Spaak [26]. However, such effects are highly dependent on the species in question, food availability, quality and size of the particles. For example, in *Daphnia*, the addition of suspended clay < 2 μm at low food levels decreased food intake by ~60–70%, whereas clay particles < 1 μm had no effect and no response to any clay addition was observed at high food levels [24]. Therefore, the animal performance and the projected effects will vary, depending on the subtleties of the experimental design when testing effects of MPs.

Using a wide range of copepod taxa, Cole et al. [27] investigated the impact of spherical polystyrene beads on feeding and found no indication of prolonged gut retention when algae were present compared to an all-natural diet. Similar observations have also been reported for cladocerans [16,28–30]. Cole et al. [27] did, however, speculate that irregularly shaped secondary MPs, which are the most common in the environment, might accumulate in zooplankton guts, thereby decreasing food intake and causing physical harm. This hypothesis deserves particular attention also because the existing experimental studies on MP ingestion have almost exclusively used commercially available fluorescent microspheres with clearly defined shape, size distribution, and chemical properties [10,27,31–33], which are readily available and easy to handle. It could be argued, however, that these particles would be relevant only as primary MPs. Yet, as a consequence of weathering and degradation processes, the majority of plastic particles in the environment should have an irregular shape and variable size [34–36]. Therefore, it is questionable whether the use of uniform spherical MPs in experiments is justifiable from ecological and ecotoxicological viewpoints.

To assess the effects of chronic exposure and to explore the underlying causes of MP toxicity for zooplankton, we conducted a series of laboratory experiments using *Daphnia magna* (Strauss) as a model organism. *Daphnia* was chosen because it is a well described standard test organism in ecotoxicological studies [37]. It is also a passive filter feeder and hence is particularly susceptible to particulate exposure. In these experiments, we compared primary (spherical; PMP) and secondary (rugged; SMP) microplastics to naturally occurring inorganic particles (kaolin); all particle types had similar size ranges. To simulate environmental conditions (food quantity/quality) and induce variations in gut retention time, assimilation efficiencies, and growth, we manipulated particle: algae ratio in the incubation media. More specifically, we asked the following questions:

1. How does MP exposure affect feeding, growth, survival, and reproductive capacity of daphnids, and are these effects different in comparison to kaolin exposure?
2. Does food availability modulate the effects of MP and kaolin exposure on daphnid growth?
3. Can MPs accumulate in daphnid guts, and, if so, does gut passage time differ between SMPs and PMPs?
4. Are there any maternal effects of chronic exposure to MP? In particular, do offspring produced by MP-exposed daphnids have lower feeding activity and survivorship?

## 2. Materials and Methods

### 2.1 Study outline

**Fig 1:**
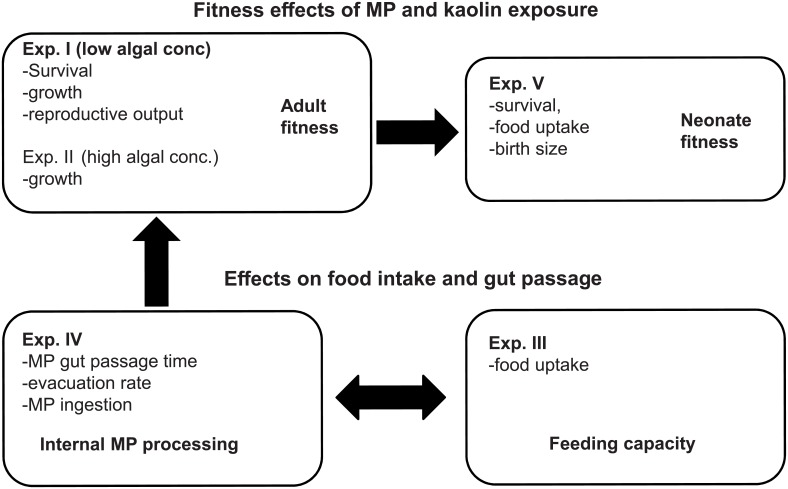
Study outline. Study outline showing the linkages between the experiments. In Experiments I-II and V, the effects of MP and kaolin exposure on fitness were studied in adults (Exp. I-II) and their offspring (Exp. V). In Experiments III-IV, food intake and MP behavior in the gut were examined and used to explain the MP effects on growth and reproduction observed in Experiments I-II and V.

We conducted five experiments (Table 1) to study the effects of particle exposure and their underlying causes (Fig 1). In Exp. I, we examined the effects of chronic exposure on basic life-history parameters (i.e., survival, growth, age at first reproduction, time between broods, number of broods produced, and total offspring number) at four concentrations of particles. The experiment was designed using the standard OECD 21-d *Daphnia* reproduction test [38], albeit at lower food concentration than recommended by the guidelines (10-fold lower). The lower food level was used in order to simulate oligotrophic systems which have been shown to contain high concentrations of inorganic particulates [39] and plastics [36]; thereby allowing us to mimic an environmentally relevant “worst case scenario”. Once the effects of long-term MP and kaolin exposure were established, a follow up experiment was conducted (Exp. II), where daphnid growth was measured at high algal concentration and MP/kaolin contribution comparable to the one used in Exp. I. The main purpose of Exp. I and II was to test whether the growth effects were related to food scarcity or to the particle effects at different feeding regimes. Further, to examine whether the observed growth effects were related to differences in food intake, the consumption of algal cells was measured in Exp. III using both MP- and kaolin-exposed daphnids. Moreover, we also assessed the potential of MPs (PMP and SMP) to form aggregates and cause obstruction in the digestive tract by measuring the amount of ingested MPs, their size distribution in the gut, gut passage time (GPT) and evacuation rate (Exp. IV). Finally, since malnutrition of mothers can result in poor offspring quality and activity, the effects of maternal exposure on the offspring feeding activity, birth size and survival were addressed in Exp. V (Fig 1).

**Table 1:** Overview of the experimental designs for experiments I-V.

Experiment	MP conc. (# mL^-1^)	% MPs of total particles	Algal conc. (μg C mL^-1^)	Endpoints	Duration of experiment	# Replicates treatment^-1^
I	10^2^	0.27	0.4	Survival, reproductive output, body size, number of broods, time between broods, age at first reproduction	21 d	10
''	10^3^	2.7	''	''	''	''
''	10^4^	21.6	''	''	''	''
''	10^5^	73.4	''	''	''	''
II	3 x 10^4^	3.5	9	Body size	10 d	7
III	2.25 x 10^5^	21.6	9	Ingestion of algae	72 h	5 (each replicate = mean of five ind.)
IV	10^4^	1.2	9	Gut passage time, evacuation rate, abundance of SMP aggregates in the gut, MP content in the gut	12 min	50
''	3 x 10^4^	3.5	''	''	''	''
V	N/A	N/A	4 (feeding test) 0 (survival)	Birth size, survival of starved and fed offspring, ingestion of algae	24 h	10 (feeding test) 6–26 (survival)

The table shows the concentration and proportion of inert particles (microplastics [MPs] and kaolin) by particle count, algal concentration, measured endpoints, the duration and the number of replicates used in each experiment.

### 2.2 Test organism

The daphnids originated from a single clone (environmental pollution test clone 5, the Federal Environment Agency, Berlin, Germany). They were cultured in 2 L of M7 medium (OECD standards 202 and 211) at a stock density of ∼20 individuals in each beaker and fed three times a week with a mixture of *Pseudokirchneriella subcapitata* and *Scenedesmus subspicatus*. No ethical approval is required for invertebrate use in toxicity testing.

### 2.3 MP preparation

As PMPs, spherical fluorescent, plastic beads ranging 1–5 μm with a mean of 4.1 ± 1.0 μm (mean equivalent spherical diameter, ESD) and a density of 1.3 g cm^-3^ (Cospheric LLC, Goleta, USA) were used. Secondary MPs were prepared by grinding 1 mm fluorescent polyethylene beads with a density of 1.0 g cm^-3^ (Cospheric LLC, Goleta, USA) in liquid nitrogen using a Retsch cryomill (Retsch, Düsseldorf, Germany). Following the grinding, the particles were sieved through a steel mesh (63 μm) to remove the largest size fraction. Due to the irregular shape, only smaller particles were able to pass the sieve resulting in a size distribution close to that of the PMPs 2.6 ± 1.8 μm ESD). Kaolin particles (Sigma-Aldrich) were slightly larger than the plastics (4.4 ± 1.1 μm ESD) and had a higher density (2.6 g cm^-3^). The size distribution of MPs and kaolin was measured using a Spectrex laser particle counter, model PC-2000 (Spectrex, Redwood city, USA).

Stock suspensions were prepared by soaking the dry MPs and the kaolin in Milli-Q water with a small addition of surfactant (Tween 80, Sigma-Aldrich, 0.01%) followed by a 2 s sonication in an ultrasonic bath to ensure disaggregation. The mixture was then allowed to soak for a minimum of 2 d according to the supplier’s recommendations; the suspensions were re-sonicated prior to use. The resulting MP-concentrations in stock suspensions were determined by direct counts using hemocytometer.

### 2.4 Life-table experiment (Exp. I)

The effects of MP and kaolin exposure on life history parameters were tested in a 21-d reproduction test according to OECD guidelines [38] at four test concentrations ranging from 10^2^ to 10^5^ particles mL^-1^ (Table 1). These concentrations were equivalent to ~0.3–73% of the total particle content in the exposure medium on a particle count basis. Reliable estimates of environmental MP concentrations in this size range and acute toxicity data are currently lacking [10] but environmental concentrations for larger MPs are generally low. For example, Kang et al. [40] found 0.02 MPs mL^-1^ (> 50 μm) in the water column of Geoje Bay, South Korea and Gorokhova [41] a maximum concentration of 0.0075 MPs mL^-1^ (> 90 μm) in the Baltic Sea proper. Considering that weathering and fragmentation most likely leads to higher concentrations of the smallest particles, we chose to work with concentrations spanning the range from suspected environmental levels to very high and unlikely to occur in nature. This ensured measurable ingestion under the experimental conditions and an overlap with naturally occurring clay particle concentrations [42,43].

Neonates (<24 h) were incubated individually in 50 mL M7 medium at 22°C under a 16:8 light dark cycle and fed 0.4 μg C mL^-1^ day^-1^ of the micro alga *Pseudokirchneriella subcapitata*. The test medium was fully replaced three times per week. The offspring number and adult mortality were recorded daily. We also recorded age at first reproduction, number of broods, average time between the broods, and adult size at the end of the experiment.

### 2.5 Effects of MP and kaolin on growth at high algal concentration (Exp. II)

Daphnia neonates (<24 h) were placed individually in glass beakers containing 50 mL M7 and fed a mixture containing 9 μg C mL^-1^ of algae (*P*. *subcapitata*) and either kaolin or MPs. This algal concentration is above the incipient limiting concentration, ILC [44]. The animals were exposed for 10 d with a complete renewal of the test medium including algae after 5 d; the food levels were estimated to stay above ILC during the entire exposure, ensuring constant filtration rates [45]. The experiment was conducted under the same light and temperature conditions as in Exp. I. The treatments included exposure to PMPs, SMPs or kaolin at 3 × 10^4^ particles mL^-1^ (3.5% by total particle count); the control daphnids were fed only algae. In each treatment and in control, 7 individuals were used as replicates. At the termination of the incubation, the daphnids were preserved in 70% EtOH for length measurements that were used for individual growth analysis (see section 2.9 for details).

### 2.6 Food intake by adult Daphnia in the presence of MPs and kaolin (Exp. III)

In this experiment, the effects of MPs and kaolin on the ingestion of algae were tested. Prior to the exposure, neonates (< 24 h) were fed with 2 μg C mL^-1^ of *P*. *subcapitata* every other day for 6 d, after which 5 individuals were exposed to either MPs or kaolin at 2.25 × 10^5^ particles mL^-1^ (21.6% by particle number) in the presence of 9 μg C mL^-1^ of *P*. *subcapitata*. The exposure was conducted in 610 mL bottles; the top of the bottles was covered with food grade cling film before the screw cap was secured. The bottles were placed on a plankton wheel and rotated at 0.5 rpm for 72 h in complete darkness to avoid algal growth during the experiment. Food intake was determined fluorometrically using a FLUOSTAR Optima microplate reader (BMG Labtech, Offenburg, Germany). See S1A Appendix for details. The feeding activity of the daphnids was assessed by measuring food intake as the difference in algal fluorescence in the feeding suspension between the start and the end of the experiment; these values were converted to carbon content using an empirically derived fluorescence to carbon content relationship (S1A Appendix).

### 2.7 MP processing in the gut (Exp. IV)

The MP-content, expressed as the integrated area of MPs in the guts, as well as size distribution were measured to characterize MP processing in the animal gut. Since SMPs were observed to form aggregates, we also assessed the number of these aggregates in the gut. An aggregate was operationally defined as a coherent visible agglomerate > 80 μm^2^.

We used a relatively low contribution of MPs to minimize the re-ingestion of particles during the gut depuration phase. Five-day old *D*. *magna* were exposed to either PMPs or SMPs at two concentrations (10^4^ and 3 × 10^4^ particles mL^-1^; equivalent to 1.2 and 3.5% of the total particle concentration in the medium) in M7 medium (500 mL) containing 9 μg C mL^-1^ of *P*. *subcapitata*. After a 1-h feeding period, 10 individuals from each experimental incubation were gently sieved, washed in M7 medium and transferred to 100 mL beakers with algal suspension at the same concentration as during the exposure, and incubated for 0, 3, 6, 9 and 12 min to clear their guts. At each time point, the animals were transferred to Eppendorf tubes using a Pasteur pipette. The excess water was then removed and the animals were preserved in 70% EtOH for fluorescence microscopy and quantification of MPs and aggregate occurrence in the gut using image analysis (S1A Appendix).

### 2.8 Maternal effects (Exp. V)

The neonates produced in Exp. I were placed individually into 96-well microplates. When possible, each brood was equally split between the treatments. The treatments were *starved animals*: a plate containing 0.25 mL well^-1^ M7 medium, and *fed animals*: M7 medium with 4 μg C mL^-1^ of algal suspension. All plates were incubated in darkness for 24 h. Upon termination of the experiment, the following endpoints were measured: (i) mortality, (ii) length of the starved neonates, and (iii) food consumption by the fed animals (as described in section 2.6).

### 2.9 Data handling and statistics

To account for differences in individual mortality between the treatments in Exp. I, we standardized the variables *reproductive output* (neonates per individual days survived, NID) and *number of broods produced* (broods per individual days survived, BID) (c.f [46]).

To visualize the differences between treatments and controls, the endpoints from Exp. I and Exp. II were expressed as z-scores, i.e., normalized to the mean of the respective control as: $Z=\left(\alpha -{\bar{x}}_{control}\right)/S.D.$, where α is the observed value and *S*.*D*. the standard deviation of the sample population.

The potential influence of the daphnid size on MP/algal ingestion, and processing in the gut was accounted for by either standardizing the response variable by individual dry weight (*DW*, Exp. IV –GPT calculations) or by including *DW* as a covariate in the statistical models (Exp. IV and V). Dry weights were estimated using the body length measured at the end of the experiments as a distance from the center of the eye to the base of the apical spine, using the length-weight relationship: *Ln*(*DW*) = 1.468+2.83×*Ln*(*L*) for daphnids, where *DW* = dry weight in μg and *L* = body length in mm [47].

To examine MP behavior in *Daphnia* guts (Exp. IV), the gut passage time and evacuation rate were estimated by fitting an exponential decay function to the MP integrated pixel area obtained by image analysis: *Y*_*t*_ = (*Y*_0_−*b*)exp(−*kt*)+*b*, where *Y* is weight-specific integrated MP area in the gut relative to the average MP area of the control (%) at time *t* (min), *b* is the plateau, and *k* is the rate constant. Gut passage time of MPs was assayed as the half-life for specific MPs (c.f. [48]).

To test for the differences in measured responses (Table 1) between the treatments or groups, we used generalized linear models (GLMs, all tests outlined in S1 Table). The type of distribution used in the models was chosen to appropriate the data. When standard distributions did not normalize residuals sufficiently, we used Box-Cox transformation [49]. Residuals were checked visually and homogeneity of variances was assessed using Bartlett’s tests [50]. Not significant interaction terms were dropped from the models and followed by separate univariate tests.

In Exp. I, we tested models for NID, BID, age at first reproduction and time between broods as a function of treatment (MPs and kaolin), Concentration (particle concentration) and the Treatment × Concentration interaction. In these models, we evaluated treatment effects on the dose-response relationship. The 50% effect concentration (EC_50_) for NID was calculated using a log-logistic model with negative binomial error structure and Bayesian inference (details in [46]). The average integrated responses per treatment and across all test concentrations were summarized in a radar plot using values normalized to the control (zero mean, unit variance).

To test whether food availability modulated the growth response to MPs or kaolin exposure, Algal Concentration, Treatment and their interaction were used as fixed variables and normalized DW as the response variable (Exp. I and II). To adjust for possible differences in the exposure time (and hence body size) between the experiments, the daphnid DW was converted to z-scores as described above. Treatment-specific differences in the food intake (Exp. III) were tested using Treatment as a single fixed predictor and DW as the response. In this comparison, only the treatments with similar contributions of the inert particles (2.7 vs. 3.5% of total particle count in Exp. I and II, respectively) were used.

In Exp. IV, the differences in the absolute amount of internalized MPs and the frequency of internalized aggregates between the treatments were tested using integrated pixel area and the frequency of aggregates in individual samples as the response variables, respectively. Treatment was used as a fixed predictor, and DW as a covariate. In the gut evacuation analysis (Exp. IV), significant outliers were identified by ROUT test and removed [51].

To test for the differences in neonate feeding ability across the treatments (Exp. V), the food intake was modeled as a function of treatment and test concentrations administered to the mothers; DW was used as a covariate. In the same way, the treatment effect on the neonate survival and size at birth were tested but without the inclusion of neonate DW as a covariate. Half–life estimates and rate constants were calculated using the non-linear regression module in GraphPad 6.0 (Prism 6.04 for Windows). All other statistical analyses were performed in R 3.1.0 [52]. Results were deemed statistically significant at α = 0.05.

## 3. Results

### 3.1 Life table experiment

**Fig 2:**
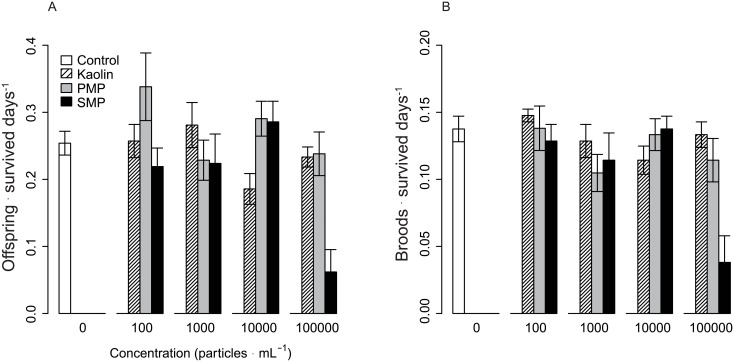
Reproductive effects. Total reproductive output (A) and number of broods (B) standardized to the number of survived days (Exp. I). In the controls, daphnids were incubated with only algae. PMP and SMP are primary and secondary MPs, respectively. Bars denote mean value ± 95% CI.

There was a significant particle type × concentration interaction for NID and BID (χ^2^_2,121_ = 17.9, p = 0.0001 and χ^2^_2,121_ = 18.0, p = 0.0001, respectively) indicating that the dose responses differed between the particle types. In the SMP-treatment, NID was approximately 76% and BID 71% lower compared to all other treatments and showed significant dose responses (S2 Table). Moreover, the SMP-exposed animals displayed a high level of mortality at the highest concentration (50% by day 14), which was not observed in either PMP or kaolin treatments (Fig 2 and S2 Table).

The EC_50_ for NID differed between the particle types, with values for SMP being approximately three times lower compared to the other particle types. The difference between SMP and kaolin was significant, whereas no significant difference between kaolin and PMP was found (S3 Table).

**Fig 3:**
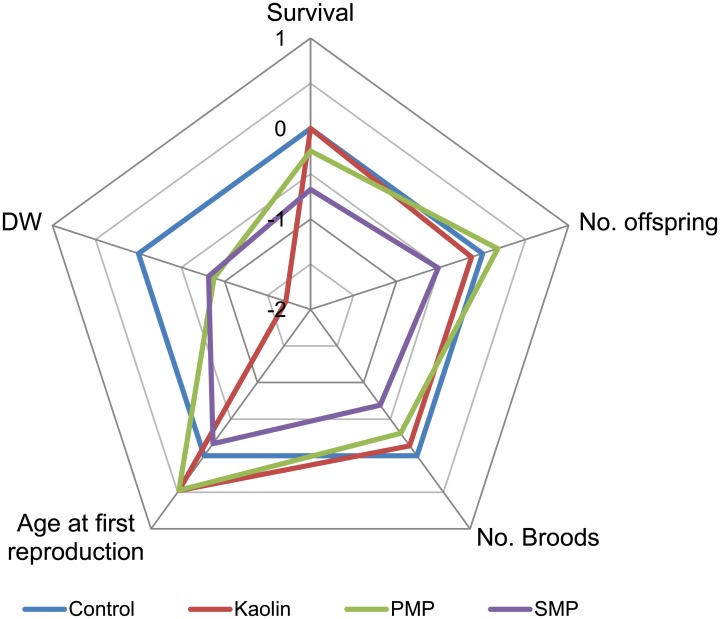
Integrated fitness responses. Radar plot of the life history traits from Exp. I averaged across all test concentrations and z-score normalized to the control (zero mean, unit variance). Positive values denote a positive response on fitness. Reciprocal transformation was applied to *Age at first reproduction* so that positive values correspond to earlier reproduction compared to the control.

No significant dose response was observed for age at first reproduction, time between broods and DW (Fig 3 and S4 Table). When averaged across all test concentrations, DW was significantly affected by MPs (S2 Table), with slower growth in all particle treatments compared to the control (24, 12, 10% lower, kaolin, PMP and SMP respectively).

### 3.2 Individual growth at high vs. low algal concentrations

**Fig 4:**
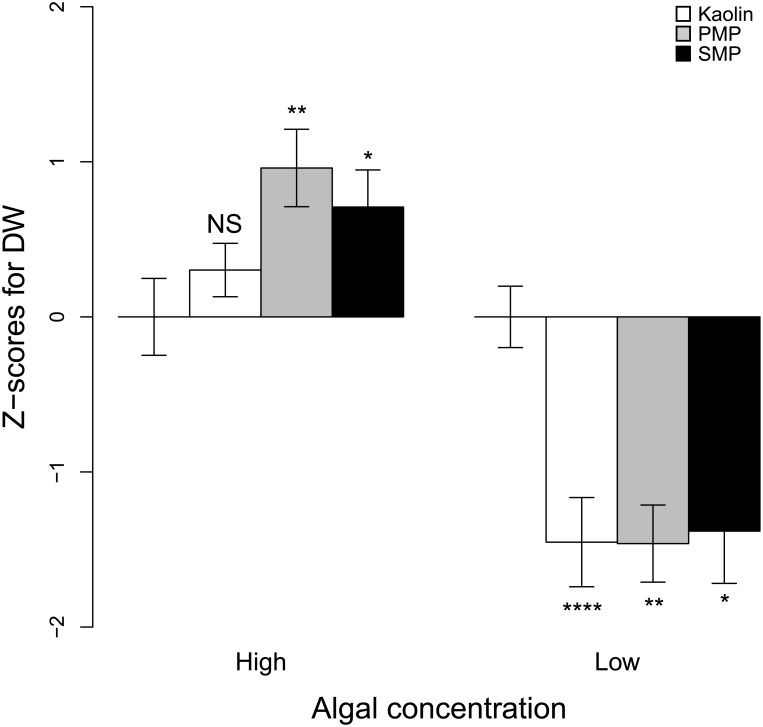
Growth at different food levels. Individual dry weight (DW) of daphnids normalized to the control (z-scores, zero mean, unit variance) at low (0.4 μg C mL^-1^, Exp. II) and high (9 μg C mL^-1^, Exp. IV) algal concentrations. Bars denote average difference from their respective controls ± 95% CI. NS = no significance, * = p < 0.05, ** = p < 0.01, **** = p < 0.0001.

The treatment effects on growth were significantly different between high and low algal concentrations (GLM, t_1,105_ = -7.8, p < 0.0001). At the low food concentration (Exp. I; Fig 4 and S5 Table), growth was lower in all treatments compared to the control: by 16% in kaolin and PMP (p < 0.001 and p = 0.008, respectively) and by 15% in SMP (p = 0.012). At the high algal concentration (Exp. II; S5 Table), significantly higher growth was observed in the PMP (by 58%; p = 0.006) and SMP (by 43%; p = 0.04) treatments compared to the control; the increase (by 18%) in the kaolin treatment was not significant (p = 0.37).

### 3.3 Algal consumption by adult *Daphnia* in the presence of MPs and kaolin

**Fig 5:**
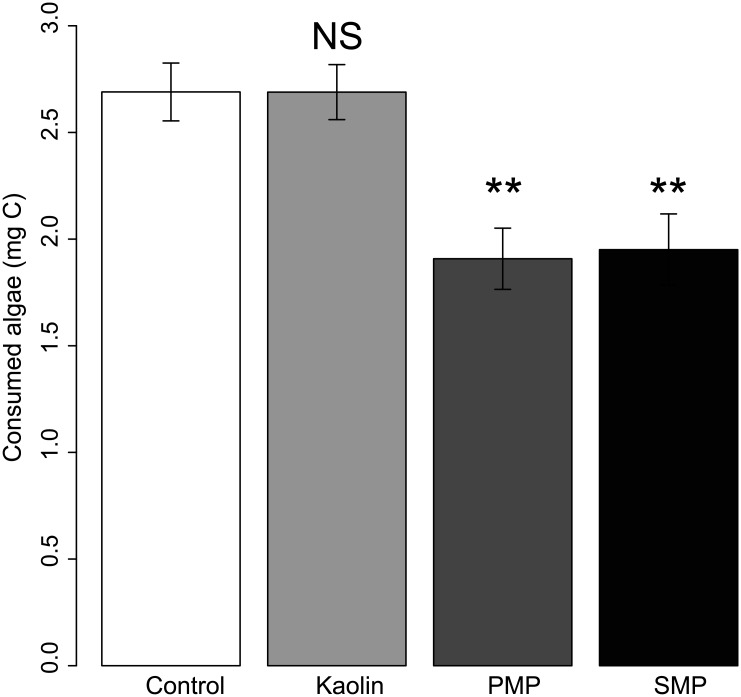
MPs decrease food consumption. Total food intake of daphnids incubated in feeding media containing algae without (control) or with addition of either spherical microplastics (PMP), ground microplastics (SMP) or kaolin. Bars denote average carbon consumption (mg C) over 72-h ± 95% CI. NS denotes non-significant difference compared to the control and ** indicates p ≤ 0.01.

MPs had a significant negative effect on the consumption of algae. Total food intake by carbon was 29% and 28% lower in the PMP and SMP treatments compared to the control and kaolin (GLM, t_3,16_ = -3.82, p = 0.002 and t_3,16_ = -3.61, p = 0.002, PMP and SMP respectively). No significant difference in food intake was observed between either PMP and SMP treatments (Tukey HSD, p = 1.0) or between kaolin and control (GLM, t_3,16_ = 18.6, p = 1.0, Fig 5).

### 3.4 Gut retention of MPs and aggregate formation

**Fig 6:**
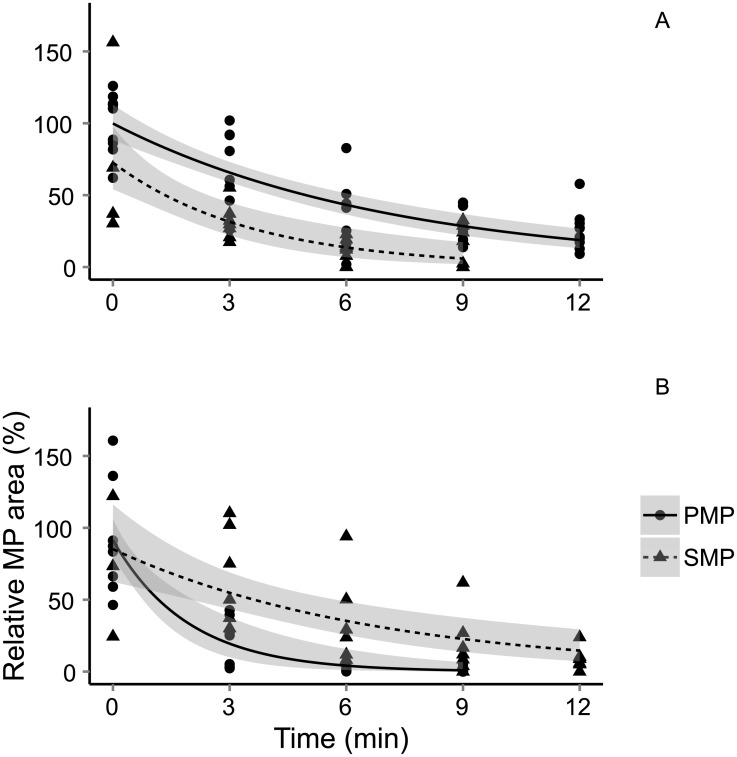
MP processing in the gut. Relative weight-specific integrated microplastic (MP) particle area in the gut as a function of time. The values are relative the average gut particle area at t = 0. Gut evacuation measured at (A) Low (10^4^ MPs mL^-1^) and (B) High (3 × 10^4^ MPs mL^-1^) particle concentrations. Grey bands represent 95% CI.

More than 90% of MPs were evacuated from the daphnid guts after 9–12 min of depuration (Fig 6). In the PMP treatment, the half-life estimates differed between the MP concentrations, with > 70% lower values at the high compared to the low concentration (1.3 and 5.0 min, respectively). In the SMP treatment, the opposite was observed, with > 80% higher values at the high concentration (2.2 and 4.3 min, for the low and high MP concentrations, respectively); these differences were statistically significant (S6 Table).

The total amount of ingested MPs was dependent on the MP-type, with 77% lower amount of SMPs in the gut compared to PMPs (GLM, t_1,135_ = -6.2, p <0.0001). The two particle types had different capacity to form aggregates, with aggregation being observed for SMP but not PMP (S1 Fig). The number of aggregates formed was positively dependent on daphnid size (GLM, Z_1,74_ = 4.1, p <0.0001), and a ~4-fold higher number of the aggregates was observed in the gut of animals exposed to the high concentration compared to the low concentration (GLM, Z_1,74_ = 4.87, p <0.0001). Moreover, the size distribution of the aggregates differed between the SMP concentrations, being highly skewed towards the smaller size range (inter quartile range, IQR = 21 μm^2^) in the low concentration and more even distribution (IQR = 27 μm^2^) in the high concentration (S2 Fig).

### 3.5 Maternal effects on offspring survival and feeding efficiency

Offspring survival over 24 h was generally high (92–100% across all concentrations and particle types) but neither survival, algal consumption or offspring body size differed significantly among the groups (S7 Table).

## 4. Discussion

### 4.1 Life history traits and particle toxicity

No significant effects on the life history traits of daphnids exposed to primary MPs or kaolin were detected. By contrast, secondary MPs did exert negative responses in reproduction and survival, albeit at very high particle abundance (10^5^ particles mL^-1^) and relative contribution to the suspended material (74%). Moreover, SMPs were found to form aggregates in daphnia guts. Given the observed aggregative properties of SMPs, it is possible that the formation and passage of aggregates through the gut might have caused some internal damage and, thereby, contributed to the elevated mortality. Microvillial damage by nanofullerene aggregates has for example been documented in *Chironomus riparius* larvae which affected both food consumption and growth negatively [53]. The possibility of such mechanism is also supported by the fact that the negative effect on algal ingestion was of equal magnitude in the presence of both plastic types (Fig 5), despite the lower absolute intake of SMPs. Thus, these microplastics could have induced some additional effects on feeding that cannot be explained by particle dilution alone.

Mortality and decreased population growth have been observed for daphnids exposed to clay particles, albeit at concentrations ~50-fold higher than our highest concentration (1 mg L^-1^) [22,25,54]. The effects of clay exposure can, however, depend on the mineral type. For example, montmorillonite, which is an expansive clay mineral, was more toxic for *D*. *magna* than both natural clay and kaolin, which indicates that seemingly similar mineral particles may have different capacity to cause damage when ingested [25]. With this in mind, it stands to reason that the inherent properties of the SMP particles where responsible for the elevated mortality. Moreover, as SMP and PMP were made of different polymers, we cannot rule out the possibility that the plastic additives or the amount of unbound and potentially toxic monomers also differed between the particles, which could have contributed to these effects. This is, however, less likely since, leachates from commercial plastic consumer products have not been shown to be toxic to *D*. *magna* even at concentrations three orders of magnitude higher than our highest concentration [55].

### 4.2 Effects on food consumption and growth in adults

At 22% of MPs in the feeding suspension, the intake of algal food by adult daphnids was reduced by ~30%. Moreover, the ingestion of MPs differed between the MP types, with ~80% lower intake of SMPs compared to PMPs. Hence, although the food intake was reduced to the same extent, the mechanisms of this reduction are likely to be different (see section 4.3).

In daphnids, both decreased feeding rates in the presence of clay and lack of effects, especially for very small (<1 um) particles or at low concentrations (10 mg L^-1^) have been reported [22,24]. In our experiments, the highest clay (kaolin) concentration was ten times lower than the no-effect concentration reported by Kirk and Gilbert [22] and Kirk [24], which can explain the observed lack of response in kaolin treatments.

When daphnids were exposed to MP/kaolin at low algal concentration for 21 d, reduced growth was observed for all particle types (Exp. I). By contrast, at high algal concentrations, the same particles exerted a positive growth response (18–58%), which was particularly high and significant for MPs (Exp. II). This suggests that growth effects mainly depend on overall food availability, which is in line with other studies demonstrating both positive and negative effects of natural particulates on the growth of filter feeders. At low food levels, Arruda et al. [56] and Rellstab and Spaak [26] observed natural clay to positively affect daphnid growth, and the authors suggested that adsorbed organic matter and bacterial biofilm on the clay surface may provide a complementary nutrition to these animals. In other studies with low food abundance, negative effects have been mainly linked to decreased energy intake [42]. The observed growth decline in the low-algae environment of Exp. I is, thus, in line with the observations of McCabe and O’Brien [42]. It can be also noted that in our study, the media used to conduct the experiment and to culture the algae as well as glassware were autoclaved. Therefore, no substantial biofilm growth would be expected during the incubation, which may explain the lack of positive effects in the kaolin- and MP-exposed daphnids. The growth stimulation by the particle addition at the high algal concentrations (Exp. II) is more difficult to explain. However, a possible mechanism could be related to low assimilation rates for algae at high food levels and a phenomenon known as superfluous feeding [57]. Urabe and Watanabe [58] showed that gross growth efficiency for *D*. *galeata* peaked at intermediate food concentrations (0.25 mg C L^-1^) and declined at the higher concentration (2.5 mg C L^-1^). Since our algal concentration was much higher than this optimum, it is very likely that the addition of microparticles decreased the efficient food concentrations to levels sustaining optimal assimilation efficiency, which improved growth.

The discrepancy between kaolin effects on feeding and growth observed in experiments I, II and III may seem counterintuitive; indeed, kaolin did not affect food intake but improved growth. However, comparing the magnitude of effects between the particle types in Exp. I and II (Fig 4) it becomes evident that the effects of kaolin on growth seem to be time dependent and increasing over time, i.e., weaker growth compared to MPs in Exp. II and equally slow growth in Exp. I, which indicates some level of adaptation to the environment.

In fact, although generally considered passive filterers, some selectivity by daphnids based on particle size [59], food quality [60] and prey hardness [15] has been reported. Mechanistic explanations to these observations vary but include adjustment of the filtering mesh and screen sizes [61]. Due to the lack of acclimation in our experiments, it is possible that kaolin was selected against due to morphological constraints of the filtering apparatus during the short term feeding experiment, while morphological adaptations had time to develop in the other experiments resulting in similar responses for all particle types [62].

### 4.3 MP processing in the digestive tract

As concerns have been raised regarding the potential of MPs to cause a mechanical obstruction of the digestive tract in zooplankters [27], we hypothesized that the ingestion of irregular SMPs would result in longer GPT. Both primary and secondary plastic particles were however evacuated within previously reported time ranges for spherical plastic particles and algae (c.f. [63,64]). However, in addition to the differences in aggregate formation, the primary and secondary MPs were significantly different in their evacuation rate from the gut. Moreover, a change in the proportion of MP in the feeding suspension caused an opposite response in the evacuation rate between SMPs and PMPs. When the proportion of MPs in the feeding suspension was low, the SMPs were evacuated faster than the PMPs. However, when this proportion—but also the total particle count in the suspension—increased 3-fold, the opposite was observed (S6 Table). For the PMPs, the increased evacuation at higher proportion of plastics in the media could be explained by the relatively higher contribution of non-digestible particles in the feeding suspension which has been shown to increase evacuation rate in daphnids [65]. Alternatively, this response can be driven by the increase in the total particle abundance and corresponding increase in gut evacuation as shown for normal daphnia feeding response to increased algal abundance [63,66]. The opposite pattern seen in the SMP treatment does however indicate other mechanisms at play. In fact, decreased evacuation at higher plastic contribution was observed in concert with the higher proportion of SMP aggregates in the guts, suggesting that the ingestion of such aggregates or MP agglomeration in the digestive tract slows down their egestion. Even though we could not detect obstruction of the digestive tract by SMPs, we did observe a clear negative effect on evacuation rate which also was reflected in negative fitness responses different from both spherical MPs and natural kaolin.

### 4.4 No maternal effects on food intake, survival and birth size

Maternal effects have been observed by, for example, Garbutt and Little [13] who found reduced clearance rates over the course of a 24 h experiment in offspring born by food limited mothers. Also, Gliwicz and Guisande [12] observed survival time during starvation to be conditional on the feeding history of the mothers, i.e. increased neonate survival if their mothers were exposed to low food levels. The F_1_ females in our study were all reared under food limited conditions and the addition of MPs and kaolin further increased this stress, which was expressed by decreased growth, fecundity and survival; especially at the highest SMP concentration. However, contrary to our expectations, no significant differences in feeding rate, birth size or survival were detected in neonates born by females exposed to MPs and kaolin.

## 5. Summary and Conclusions

Daphnid life history responses to the plastic particles in the size range 2–5 μm and MP contribution to the feeding suspension <73% do not differ significantly from those exerted by natural inert particles, such as kaolin. At extremely high concentrations, the secondary microplastics may, however, be harmful. The fact that occurrence of aggregates and gut evacuation rate were affected differentially depending on the plastic type suggests that capacity to hinder feeding and food processing may differ between the primary and secondary MPs. This has implications for both the experimental design when studying MP-effects in biota and the risk assessment of MPs in the environment. The propensity of secondary MPs to form aggregates indicates that primary MPs, i.e. commercially available microplastic beads, cannot be used to generalize the effects of MPs in feeding studies. Moreover, since a passive ingestion of inert microparticles is more dependent on the particle: algae ratio than the MP concentration *per se*, we need to acknowledge that these concentrations must be viewed in relation to available food and that MP effects may vary depending on the feeding environment. To adequately assess the potential hazards of MPs, carefully designed experimental studies with varying MP contribution to the exposure media, polymer types, particle sizes and shapes are needed.

## Supporting Information

S1 Appendix Fluorescence measurements, carbon to particle number conversions and image analysis. (DOCX)

S1 Fig MP ingestion by *D*. *magna*. Microphotographs of primary and secondary microplastics (PMP and SMP respectively) used in the experiment and *Daphnia magna* after the feeding trials: (A) PMP particles; no visible agglomeration; (B) SMP particles; substantial MP/MP-aggregate formation is observed in the feeding suspension (encircled); (C) *Daphnia* fed PMP particles in Exp. I. The particles are visible as green dots in the gut-area; (D) *Daphnia* fed SMP particles. MP/MP-algae aggregates are visible in the gut (encircled). The absolute amount of SMPs in the gut is also considerably lower compared to the daphnids fed PMPs even though the plastic:algae ratio was the same.
(TIF)

S2 Fig Secondary microplastic-aggregates in the gut. Density plot showing the size distribution of secondary microplastic-aggregates (SMPs) formed in the gut (Exp. IV) at two test concentrations: 10^4^ (low conc.) and 3 × 10^4^ (high conc.) MPs mL^-1^.
(EPS)

S1 Table Statistical outline for Exp. I. Summary of the generalized linear models used for the data analysis. Explanations: Treatment refers to the test particles used (kaolin, PMP and SMP) and the control; Concentration—the test particle concentrations used; Algal concentration (0.4 and 9 μg C mL^-1^); Time –the time range within which gut passage time was measured. * indicates that two separate tests were run for each MP-type (PMP or SMP). Abbreviations: NID = Number of produced offspring standardized by the number of individual survived days, BID = number of broods produced standardized by the number of individual survived days, TBB = time between broods, AFR = age at first reproduction, WSRD = weight specific relative decrease in MP content and DW = dry weight.
(DOCX)

S2 Table GLM results for Exp. I. GLM results for the life-history parameters measured in Exp. I as a function of concentration and treatment. The estimate, shows the difference from the control. Significant effects are in bold face. Abbreviations: NID = Number of produced offspring standardized by the number of individual survived days, BID = number of broods produced standardized by the number of individual survived days, AFR = age at first reproduction (days), TBB = time between broods (days), DW = dry weight (μg).
(DOCX)

S3 Table EC50 values. EC_50_ ± 95% confidence intervals for the number of produced offspring standardized by the number of individual survived days (NID) for primary and secondary MPs (PMP and SMP) and kaolin. * EC_50_ is significantly lower for SMP compared to kaolin.
(DOCX)

S4 Table Descriptive statistics. Descriptive statistics for life-history parameters obtained from Exp. I. Values are reported as mean ± standard deviation. Offspring = cumulative reproductive output, Surv.days = number of survived days, Broods = number of broods per female, AFR = Age at first reproduction, TBB = time between broods and DW = dry weight (μg). Treatment denotes the different combinations of particles (PMP = primary MPs, SMP = secondary MPs, kaolin = kaolin clay particles and control = only algae) and concentration denotes the five concentrations used, increasing logarithmically from 0 (control) to 1 × 10^5^ particles mL^-1^.
(DOCX)

S5 Table GLM results for growth at high vs. low food. GLM table for the comparison of body size (μg DW) between high (9 μg C mL^-1^) and low (0.4 μg C mL^-1^) algal concentration. The estimate shows the deviation from the control. Significant results are in bold face.
(DOCX)

S6 Table Gut evacuation rates of primary and secondary microplastics. Rate constants (K), half-lives and their 95% confidence intervals (CI) established in Exp. IV for the primary and secondary MPs (PMP and SMP, respectively) at different particle concentrations.
(DOCX)

S7 Table GLM results–maternal effects. GLM results for maternal effects (Exp. V). Survival, size at birth and algal consumption in *Daphnia* neonates was modeled as a function of exposure concentration and particle type (primary, secondary MPs, kaolin and control). Daphnid dry weight (DW) was used as a covariate to test for differences in algal consumption. Interaction effects were also tested and found non-significant in all cases.
(DOCX)
