# Supplementary material for: The Effects of Natural and Anthropogenic Microparticles on Individual Fitness in Daphnia magna
Source: PLoS One. 2016 May 13;11(5):e0155063. doi: 10.1371/journal.pone.0155063 (PMC4866784; doi:10.1371/journal.pone.0155063)
Supplement: S1 Appendix — (DOCX) [file pone.0155063.s001.docx]

**Appendix S1: Fluorescence measurements, carbon to particle number conversions and image analysis.**

**S 1.1. Fluorescence to carbon and carbon to particle number conversions**

The carbon content of the algae in the feeding suspensions used in Exp. III was established using empirically derived standard curves run in a FLUOSTAR Optima microplate reader (BMG Labtech, Offenburg, Germany). To establish the relationship between the fluorescence and carbon content of the algae, we logarithmically diluted an algal stock suspension containing 9 µg C mL^-1^ *Pseudokirchneriella subcapitata* and either PMP, SMP or kaolin particles at 2.25 × 10^5^ particles mL^-1^ ranging 0-10^4^× dilution. The standard curve for the control contained only algae; M7 was used for the blanks and each concentration was run in triplicate. Filter wavelengths were 400-480 nm (excitation) and 640-680 nm (emission); gain = 1407. Goodness of fit for the linear relationship between carbon content and fluorescence was high (R^2^ > 0.99) for all treatments. The slope parameter *k* of the linear relationship: $C=kF-m$ where *C* = the carbon content of algae, *F* = the fluorescence and *m* = the intercept, was equal for all treatments (0.0002); only the intercepts differed; *m*_Control_ = -0.0814, *m*_SMP_ = -0.434, *m*_PMP_ = -1.744, *m*_Kaolin_ = -0.1113. The numerical concentration of algal cells *P* was estimated using the following conversion formula: $P=90785\times C$, where *P* was measured using a hemocytometer.

**S 1.2 Image analysis**

Individual *Daphnia* were photographed on their lateral side using a fluorescence microscope (Leica DMBR, Leitz, Germany) fitted with a digital camera (Canon 5D mark III) to visualize the fluorescent plastic MPs followed by image analysis using ImageJ software [67]. To calculate the amount of MPs in the guts, the image was cropped leaving only the gut for examination. To isolate the particles, color thresholding was used, and the image was transformed to black and white [68]. Subsequently, the total integrated pixel area was measured. Five daphnids that received algae but no MPs were used as blanks, and the mean integrated pixel area of MPs in the digestive tract was subtracted from the values obtained for the exposed animals. Moreover, because SMP particles were found to aggregate, we also measured the size (area) and size distribution of such aggregates in the guts using the particle counter plug-in in Image J. MP-aggregates were operationally defined as particles having an area larger than individual MPs administered in the feeding suspension, i.e. >80 µm^2^.
