# Supplementary figures and images for: The Effects of Natural and Anthropogenic Microparticles on Individual Fitness in Daphnia magna
Source: PLoS One. 2016 May 13;11(5):e0155063. doi: 10.1371/journal.pone.0155063 (PMC4866784; doi:10.1371/journal.pone.0155063)

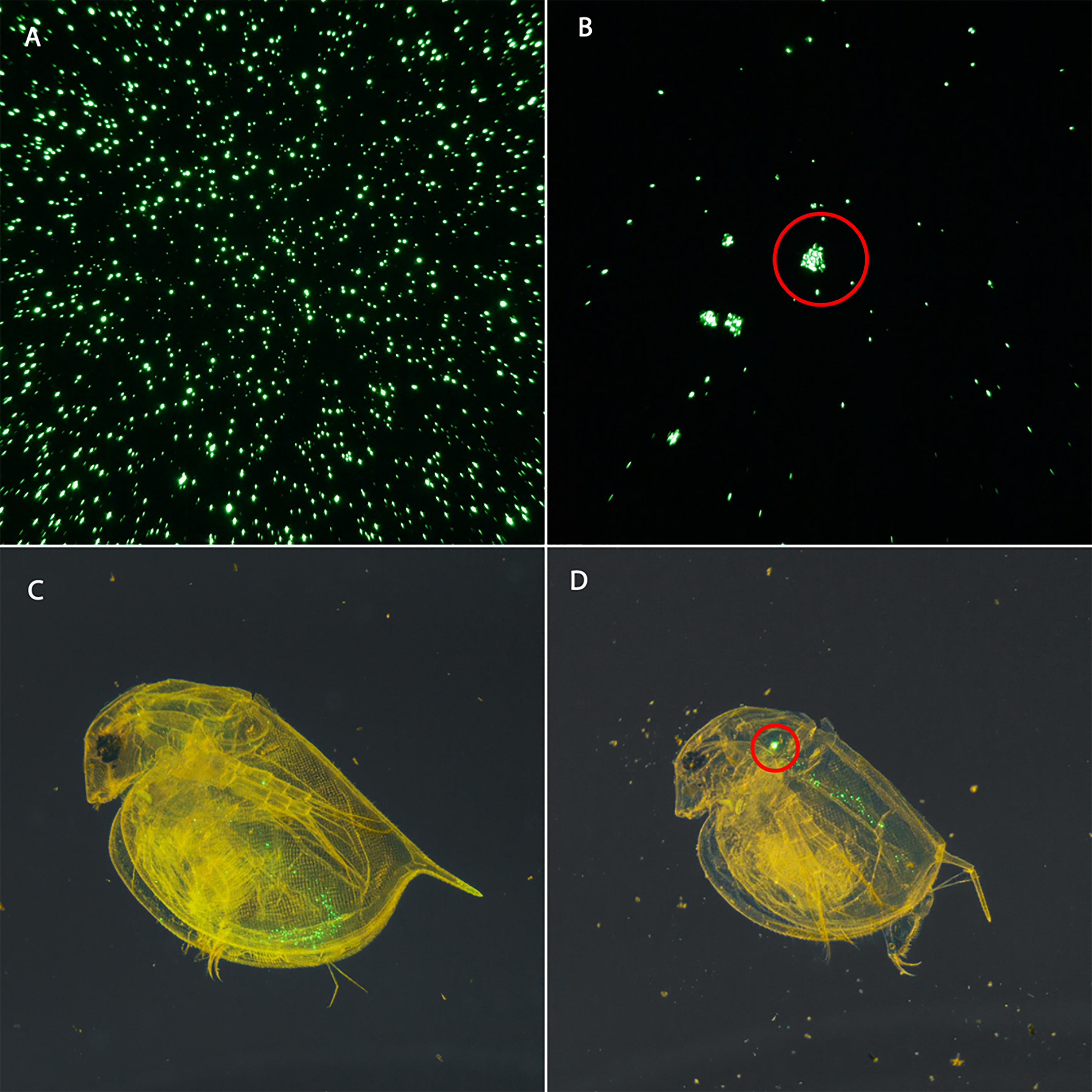

Supplement: S1 Fig — Microphotographs of primary and secondary microplastics (PMP and SMP respectively) used in the experiment and Daphnia magna after the feeding trials: (A) PMP particles; no visible agglomeration; (B) SMP particles; substantial MP/MP-aggregate formation is observed in the feeding suspension (encircled); (C) Daphnia fed PMP particles in Exp. I. The particles are visible as green dots in the gut-area; (D) Daphnia fed SMP particles. MP/MP-algae aggregates are visible in the gut (encircled). The absolute amount of SMPs in the gut is also considerably lower compared to the daphnids fed PMPs even though the plastic:algae ratio was the same. (TIF) [file pone.0155063.s002.tif]
