# Supplementary material for: The Effects of Natural and Anthropogenic Microparticles on Individual Fitness in Daphnia magna
Source: PLoS One. 2016 May 13;11(5):e0155063. doi: 10.1371/journal.pone.0155063 (PMC4866784; doi:10.1371/journal.pone.0155063)
Supplement: S1 Table — Summary of the generalized linear models used for the data analysis. Explanations: Treatment refers to the test particles used (kaolin, PMP and SMP) and the control; Concentration—the test particle concentrations used; Algal concentration (0.4 and 9 μg C mL-1); Time –the time range within which gut passage time was measured. * indicates that two separate tests were run for each MP-type (PMP or SMP). Abbreviations: NID = Number of produced offspring standardized by the number of individual survived days, BID = number of broods produced standardized by the number of individual survived days, TBB = time between broods, AFR = age at first reproduction, WSRD = weight specific relative decrease in MP content and DW = dry weight. (DOCX) [file pone.0155063.s004.docx]

**Table S1. Statistical outline for Exp. I**

| **Experiment** | **Response variable** | **Distribution** | **Predictor** |
| --- | --- | --- | --- |
| Exp. I | NID (numbers/day) | Negative binomial | Treatment |
|  |  |  | Concentration |
|  |  |  | Treatment × Concentration |
|  | BID (numbers/day) | Normal | Treatment |
|  |  |  | Concentration |
|  |  |  | Treatment × Concentration |
|  | AFR (day) | Box-Cox | Treatment |
|  |  |  | Concentration |
|  |  |  | Treatment × Concentration |
|  | TBB (days) | Box-Cox | Treatment |
|  |  |  | Concentration |
|  |  |  | Treatment × Concentration |
|  | DW (µg) | Box-Cox | Treatment |
|  |  |  | Concentration |
|  |  |  | Treatment × Concentration |
|  | Survival (yes/no) | Binomial | Treatment |
|  |  |  | Concentration |
|  |  |  | Treatment × Concentration |
| Exp. II | DW (µg) | Normal | Treatment |
|  |  |  | Algal Concentration |
|  |  |  | Treatment × Algal Concentration |
| Exp. III | Food intake (mg C) | Box-Cox | Treatment |
| Exp. IV | *WSRD (% decrease / µg) | Box-Cox | Time |
|  |  |  | Concentration |
|  |  |  | Time × Concentration |
|  | Log(MP-content in the gut [Integrated pixel area]) | Normal | Treatment |
|  |  |  | Concentration |
|  |  |  | Treatment × Concentration |
|  |  |  | DW |
|  | MP-aggregates (counts) | Poisson | Treatment |
|  |  |  | Concentration |
|  |  |  | Treatment × Concentration |
|  |  |  | DW |
| Exp. V | Food intake (µg C) | Box-Cox | Treatment |
|  |  |  | Concentration |
|  |  |  | Treatment × Concentration |
|  |  |  | DW |
|  | Survival (yes/no) | Binomial | Treatment |
|  |  |  | Concentration |
|  |  |  | Treatment × Concentration |
|  | Size at birth (DW, µg) | Normal | Treatment |
|  |  |  | Concentration |
|  |  |  | Treatment × Concentration |

Summary of the generalized linear models used for the data analysis. Explanations: Treatment refers to the test particles used (kaolin, PMP and SMP) and the control; Concentration – the test particle concentrations used; Algal concentration (0.4 and 9 µg C mL^-1^); Time –the time range within which gut passage time was measured. * indicates that two separate tests were run for each MP-type (PMP or SMP). Abbreviations: NID =Number of produced offspring standardized by the number of individual survived days, BID = number of broods produced standardized by the number of individual survived days, TBB = time between broods, AFR = age at first reproduction, WSRD = weight specific relative decrease in MP content and DW = dry weight.
