# Supplementary material for: The Effects of Natural and Anthropogenic Microparticles on Individual Fitness in Daphnia magna
Source: PLoS One. 2016 May 13;11(5):e0155063. doi: 10.1371/journal.pone.0155063 (PMC4866784; doi:10.1371/journal.pone.0155063)
Supplement: S2 Table — GLM results for the life-history parameters measured in Exp. I as a function of concentration and treatment. The estimate, shows the difference from the control. Significant effects are in bold face. Abbreviations: NID = Number of produced offspring standardized by the number of individual survived days, BID = number of broods produced standardized by the number of individual survived days, AFR = age at first reproduction (days), TBB = time between broods (days), DW = dry weight (μg). (DOCX) [file pone.0155063.s005.docx]

**Table S2. GLM results for Exp. I**

|  |  | **Concentration** | | **Treatment** | |
| --- | --- | --- | --- | --- | --- |
| **Dependent variable** | **Particle type** | **Estimate** | **p** | **Estimate** | **p** |
| NID | Kaolin | -7.7 × 10^-7^ | 0.53 | -0.06 | 0.73 |
|  | PMP | -1.6 × 10^-6^ | 0.32 | 0.08 | 0.66 |
|  | SMP | -1.3 × 10^-5^ | **0.0002** | -0.26 | 0.14 |
| BID | Kaolin | -8.5 × 10^-9^ | 0.94 | -0.01 | 0.71 |
|  | PMP | -1.4 × 10^-7^ | 0.40 | -0.02 | 0.40 |
|  | SMP | -9.2 × 10^-7^ | **<0.0001** | -0.03 | 0.06 |
| AFR | Kaolin | 3.1 × 10^-8^ | 0.98 | -0.06 | 0.56 |
|  | PMP | -6.2 × 10^-7^ | 0.60 | -0.06 | 0.56 |
|  | SMP | 2.2 × 10^-7^ | 0.90 | 0.02 | 0.85 |
| TBB | Kaolin | 6.3 × 10^-7^ | 0.71 | 0.56 | 0.14 |
|  | PMP | 2.7 × 10^-6^ | 0.12 | 0.33 | 0.39 |
|  | SMP | -3.3 × 10^-7^ | 0.92 | -0.21 | 0.57 |
| Survival | Kaolin | -6.6 × 10^-7^ | 0.18 | 5.0 × 10^-15^ | 1.00 |
|  | PMP | 3.1 × 10^-7^ | 0.70 | -4.3 × 10^-2^ | 0.60 |
|  | SMP | -2.8 × 10^-6^ | **0.002** | -1.2 × 10^-1^ | 0.14 |
| DW | Kaolin | -7.7 × 10^-8^ | 0.05 | -0.02 | **<0.0001** |
|  | PMP | -3.4 × 10^-8^ | 0.22 | -0.01 | **0.008** |
|  | SMP | 3.1 × 10^-8^ | 0.37 | -0.01 | **0.02** |

GLM results for the life-history parameters measured in Exp. I as a function of concentration and treatment. The estimate, shows the difference from the control. Significant effects are in bold face. Abbreviations: NID =Number of produced offspring standardized by the number of individual survived days, BID = number of broods produced standardized by the number of individual survived days, AFR = age at first reproduction (days), TBB = time between broods (days), DW = dry weight (µg).
