# Supplementary material for: The Effects of Natural and Anthropogenic Microparticles on Individual Fitness in Daphnia magna
Source: PLoS One. 2016 May 13;11(5):e0155063. doi: 10.1371/journal.pone.0155063 (PMC4866784; doi:10.1371/journal.pone.0155063)
Supplement: S3 Table — EC50 ± 95% confidence intervals for the number of produced offspring standardized by the number of individual survived days (NID) for primary and secondary MPs (PMP and SMP) and kaolin. * EC50 is significantly lower for SMP compared to kaolin. (DOCX) [file pone.0155063.s006.docx]

**Table S3. EC50 values**

| **Type** | **EC_50_** | **95 % CI** |
| --- | --- | --- |
| SMP* | 8.6 × 10^4^ | 3.7 × 10^4^ - 1.5 × 10^5^ |
| PMP | 2.8 × 10^5^ | 1.1 × 10^5^ - 1.0 × 10^6^ |
| Kaolin | 3.0 × 10^5^ | 4.4 × 10^4^ - 1.5 × 10^6^ |

EC_50_ ± 95% confidence intervals for the number of produced offspring standardized by the number of individual survived days (NID) for primary and secondary MPs (PMP and SMP) and kaolin. * EC_50_ is significantly lower for SMP compared to kaolin.
