# Supplementary material for: The Effects of Natural and Anthropogenic Microparticles on Individual Fitness in Daphnia magna
Source: PLoS One. 2016 May 13;11(5):e0155063. doi: 10.1371/journal.pone.0155063 (PMC4866784; doi:10.1371/journal.pone.0155063)
Supplement: S4 Table — Descriptive statistics for life-history parameters obtained from Exp. I. Values are reported as mean ± standard deviation. Offspring = cumulative reproductive output, Surv.days = number of survived days, Broods = number of broods per female, AFR = Age at first reproduction, TBB = time between broods and DW = dry weight (μg). Treatment denotes the different combinations of particles (PMP = primary MPs, SMP = secondary MPs, kaolin = kaolin clay particles and control = only algae) and concentration denotes the five concentrations used, increasing logarithmically from 0 (control) to 1 × 10^5^ particles mL-1. (DOCX) [file pone.0155063.s007.docx]

**Table S4. Descriptive statistics**

| **Treatment** | **Concentration** | **Offspring** | | | **Broods** | | | **Surv.days** | | | **AFR** | | | **TBB** | | | **DW** | | |
| --- | --- | --- | --- | --- | --- | --- | --- | --- | --- | --- | --- | --- | --- | --- | --- | --- | --- | --- | --- |
| Control | 0 | 5.3 | ± | 1.1 | 21.0 | ± | 0.0 | 2.9 | ± | 0.6 | 12.1 | ± | 1.6 | 4.1 | ± | 1.2 | 66.9 | ± | 4.3 |
| PMP | 10^2^ | 7.1 | ± | 3.4 | 19.1 | ± | 6.0 | 2.9 | ± | 1.1 | 11.0 | ± | 0.0 | 3.6 | ± | 0.6 | 56.7 | ± | 4.0 |
|  | 10^3^ | 4.8 | ± | 2.0 | 19.7 | ± | 4.1 | 2.2 | ± | 0.9 | 12.3 | ± | 2.4 | 4.7 | ± | 1.4 | 56.3 | ± | 5.4 |
|  | 10^4^ | 6.1 | ± | 1.7 | 21.0 | ± | 0.0 | 2.8 | ± | 0.8 | 11.3 | ± | 1.1 | 3.7 | ± | 1.5 | 64.3 | ± | 7.1 |
|  | 10^5^ | 5.0 | ± | 2.2 | 20.7 | ± | 1.0 | 2.4 | ± | 1.1 | 10.9 | ± | 0.6 | 5.3 | ± | 2.4 | 57.4 | ± | 7.9 |
| SMP | 10^2^ | 4.6 | ± | 1.8 | 21.0 | ± | 0.0 | 2.7 | ± | 0.8 | 12.4 | ± | 1.9 | 4.1 | ± | 0.8 | 59.2 | ± | 8.1 |
|  | 10^3^ | 4.7 | ± | 2.9 | 17.5 | ± | 7.4 | 2.4 | ± | 1.4 | 12.9 | ± | 1.5 | 3.6 | ± | 0.3 | 56.8 | ± | 6.9 |
|  | 10^4^ | 6.0 | ± | 1.9 | 21.0 | ± | 0.0 | 2.9 | ± | 0.6 | 11.8 | ± | 2.1 | 4.1 | ± | 1.2 | 59.2 | ± | 5.7 |
|  | 10^5^ | 1.3 | ± | 2.2 | 15.2 | ± | 5.1 | 0.8 | ± | 1.3 | 12.7 | ± | 2.1 | 3.8 | ± | 0.3 | 64.0 | ± | 3.9 |
| Kaolin | 10^2^ | 5.4 | ± | 1.7 | 21.0 | ± | 0.0 | 3.1 | ± | 0.3 | 11.0 | ± | 0.8 | 3.7 | ± | 0.5 | 48.8 | ± | 10.0 |
|  | 10^3^ | 5.9 | ± | 2.2 | 21.0 | ± | 0.0 | 2.7 | ± | 0.8 | 11.2 | ± | 1.5 | 5.3 | ± | 1.9 | 56.3 | ± | 6.6 |
|  | 10^4^ | 3.9 | ± | 1.5 | 21.0 | ± | 0.0 | 2.4 | ± | 0.7 | 11.9 | ± | 1.5 | 4.6 | ± | 1.7 | 50.9 | ± | 8.2 |
|  | 10^5^ | 4.9 | ± | 1.0 | 21.0 | ± | 0.0 | 2.8 | ± | 0.6 | 11.4 | ± | 1.3 | 4.7 | ± | 1.6 | 48.4 | ± | 13.9 |

Descriptive statistics for life-history parameters obtained from Exp. I. Values are reported as mean ± standard deviation. Offspring = cumulative reproductive output, Surv.days = number of survived days, Broods = number of broods per female, AFR = Age at first reproduction, TBB = time between broods and DW = dry weight (µg). Treatment denotes the different combinations of particles (PMP = primary MPs, SMP =secondary MPs, kaolin = kaolin clay particles and control = only algae) and concentration denotes the five concentrations used, increasing logarithmically from 0 (control) to 1 × 10^5^ particles mL^-1^.
