# Supplementary material for: The Effects of Natural and Anthropogenic Microparticles on Individual Fitness in Daphnia magna
Source: PLoS One. 2016 May 13;11(5):e0155063. doi: 10.1371/journal.pone.0155063 (PMC4866784; doi:10.1371/journal.pone.0155063)
Supplement: S5 Table — GLM table for the comparison of body size (μg DW) between high (9 μg C mL-1) and low (0.4 μg C mL-1) algal concentration. The estimate shows the deviation from the control. Significant results are in bold face. (DOCX) [file pone.0155063.s008.docx]

**Table S5. GLM results for growth at high vs. low food**

| **Test** | **Type** | **Estimate** | **t** | **p** |
| --- | --- | --- | --- | --- |
| Low algal conc. | Kaolin | -10.5 | -3.9 | **<0.001** |
|  | PMP | -10.6 | -3.8 | **<0.001** |
|  | SMP | -10.0 | -3.5 | **0.001** |
| High algal conc. | Kaolin | 7.55 | 0.9 | 0.37 |
|  | PMP | 24.0 | 2.8 | **0.006** |
|  | SMP | 17.7 | 2.1 | **0.04** |

GLM table for the comparison of body size (µg DW) between high (9 µg C mL^-1^) and low (0.4 µg C mL^-1^) algal concentration. The estimate shows the deviation from the control. Significant results are in bold face.
