# Supplementary material for: The Effects of Natural and Anthropogenic Microparticles on Individual Fitness in Daphnia magna
Source: PLoS One. 2016 May 13;11(5):e0155063. doi: 10.1371/journal.pone.0155063 (PMC4866784; doi:10.1371/journal.pone.0155063)
Supplement: S6 Table — Rate constants (K), half-lives and their 95% confidence intervals (CI) established in Exp. IV for the primary and secondary MPs (PMP and SMP, respectively) at different particle concentrations. (DOCX) [file pone.0155063.s009.docx]

**Table S6. Gut evacuation rates of primary and secondary microplastics**

| **Type** | **Concentration** | **K** | **95% CI** | **Half-life** | **95% CI** |
| --- | --- | --- | --- | --- | --- |
| PMP | 10^4^ | 0.14 | 0.11 – 0.16 | 5.02 | 4.21 – 6.20 |
|  | 3 × 10^4^ | 0.54 | 0.32 – 0.76 | 1.29 | 0.92 – 2.20 |
| SMP | 10^4^ | 0.31 | 0.21 – 0.41 | 2.23 | 1.69 – 3.27 |
|  | 3 × 10^4^ | 0.16 | 0.11 – 0.21 | 4.29 | 3.26 – 6.26 |

Rate constants (K), half-lives and their 95% confidence intervals (CI) established in Exp. IV for the primary and secondary MPs (PMP and SMP, respectively) at different particle concentrations.
