# Supplementary material for: The Effects of Natural and Anthropogenic Microparticles on Individual Fitness in Daphnia magna
Source: PLoS One. 2016 May 13;11(5):e0155063. doi: 10.1371/journal.pone.0155063 (PMC4866784; doi:10.1371/journal.pone.0155063)
Supplement: S7 Table — GLM results for maternal effects (Exp. V). Survival, size at birth and algal consumption in Daphnia neonates was modeled as a function of exposure concentration and particle type (primary, secondary MPs, kaolin and control). Daphnid dry weight (DW) was used as a covariate to test for differences in algal consumption. Interaction effects were also tested and found non-significant in all cases. (DOCX) [file pone.0155063.s010.docx]

**Table S7. GLM results –maternal effects**

| **Response variable** | **Predictor** | **χ^2^** | **df** | **p** |
| --- | --- | --- | --- | --- |
| Survival | Concentration | 1.19 | 1 | 0.75 |
|  | Type | 0.61 | 3 | 0.73 |
| Size at birth | Concentration | 0.00 | 1 | 0.94 |
|  | Type | 0.88 | 3 | 0.83 |
| Algal consumption | Concentration | 0.01 | 1 | 0.93 |
|  | Type | 2.63 | 3 | 0.45 |
|  | DW | 0.00 | 1 | 0.98 |

GLM results for maternal effects (Exp. V). Survival, size at birth and algal consumption in *Daphnia* neonates was modeled as a function of exposure concentration and particle type (primary, secondary MPs, kaolin and control). Daphnid dry weight (DW) was used as a covariate to test for differences in algal consumption. Interaction effects were also tested and found non-significant in all cases.
